# Advances in doubled haploid technology for rice breeding: mechanisms, applications, and future perspectives

**DOI:** 10.3389/fpls.2025.1709033

**Published:** 2026-01-05

**Authors:** Yiling Lin, Fangming Mao, Yuheng Huang, Derun Huang, Kejian Wang, Song Yan, Chaolei Liu

**Affiliations:** 1 State Key Laboratory of Rice Biology and Breeding, China National Rice Research Institute, Hangzhou, China; 2 Jiangxi Academy of Agricultural Sciences, Nanchang, China; 3 Hainan Seed Industry Laboratory, Sanya, China

**Keywords:** DH technology, haploid inducer, haploid induction, rice, breeding

## Abstract

Production of homozygous inbred lines is crucial in rice breeding programs. Doubled haploid (DH) technology enables the efficient development of pure lines and significantly shortens the breeding cycle compared to conventional methods. This review summarizes recent advances in the four key steps of DH technology mediated by haploid inducers in rice: (1) exploration of haploid induction genes and development of high-efficiency haploid inducers, (2) rapid identification of haploids using molecular and morphological markers, (3) chromosome doubling of haploid plants through colchicine treatment, and (4) application of DH technology in rice breeding. Furthermore, current challenges and issues associated with each step are discussed. With ongoing advancements and the resolution of existing limitations, DH technology based on haploid inducers is anticipated to become a fundamental and widely adopted tool in rice breeding.

## 1 Introduction

Rice is a staple food for over half the world’s population. However, current crop yields cannot meet the expected food demand by 2050 due to population growth (Ray et al., 2013). Therefore, there is an urgent need to speed up crop breeding and develop high-yield rice varieties. DH technology, based on chromosomal elimination, produces haploid plants from heterozygous donor plants and then doubles their chromosomes to create homozygous DH lines that can be directly used in breeding. Compared with traditional methods, this technology generates genetically stable, fully homozygous breeding materials in just 1–2 generations, greatly shortening the breeding process (Widiez, 2021). Although DH technology offers considerable advantages, its application in rice breeding remains relatively limited, such as strong genetic background dependence and low haploid induction efficiency, and its full potential has not yet been fully exploited (Mayakaduwa and Silva, 2023; Qu et al., 2024).

One of the key steps in DH technology is haploid induction. Currently, methods for inducing haploids in plants can be divided into two categories: *in vitro* and *in vivo*. *In vitro* methods typically involve the culture of immature anthers or pollen under specific hormonal or stress conditions to generate haploid plants (Mayakaduwa and Silva, 2023). In contrast, *in vivo* methods rely on crossbreeding with specialized inducer lines or the application of treatments to pollen in order to produce haploid progeny (Ren et al., 2017). In rice, the *in vitro* method utilizing anther/pollen culture has contributed to the development of several *japonica* varieties, such as Zhonghua 8, Zhonghua 9, and Xin Xiu (Mishra and Rao, 2016). However, *in vitro* induction methods face multiple challenges, including high costs, strong genotype dependence (with much lower efficiency in *indica* rice), and technically demanding protocols that can result in failure modes such as albino plantlet formation (**Table 1**). Current studies demonstrate that *in vivo* haploid induction offers distinct advantages in operational simplicity, cost-effectiveness, and genotype independence (Jacquier et al., 2020). Meanwhile, *in vivo* strategies employing haploid inducers have gained increasing attention and are being actively explored in rice in recent years.

**Table 1:** Comparison of *in vivo* and *in vitro* induction methods in rice.

Comparison criteria	*In vivo*	*In vitro*
Efficiency ranges	0%- 12.5% Liu et al. (2024a)	Callus induction rate: *japonica* rice (10%–40%); *indica* rice (mean 0.5%) Mayakaduwa and Silva (2023)
Genotype dependence	Broad genotype applicability	*Japonica* > *Indica*Yan et al. (1996)
Failure modes	Low doubling efficiency	Chimerism; albino plantlet formation Kyum et al. (2022)
Cost	Low	High
Time	1–2 generations	1–2 generations

*In vivo* DH technology utilizing haploid inducers has been widely adopted in commercial maize breeding programs. The first documented haploid inducer in maize, Stock 6, exhibits a haploid induction rate (HIR) of 1%-3% (Coe, 1959). As the foundational line for subsequent inducer development, Stock 6 has been instrumental in advancing maize haploid breeding. Through the introgression of Stock 6 into diverse genetic backgrounds, high-efficiency inducers such as UH400, RWS, and CAU5 have been developed, achieving HIRs in the range of 6%-15% (Büter, 1997; Liu et al., 2017; Nair et al., 2020). These advances have significantly promoted the adoption of this technology in maize breeding. The genetic basis of haploid induction in maize was elucidated in 2017, with studies establishing that loss-of-function mutations in the key gene *MATRILINEAL* (*MATL*)/*NOT LIKE DAD* (*NLD*)/*ZmPHOSPHOLIPASE‐A1* (*ZmPLA1*) are responsible for triggering haploid induction. The induction rates conferred by these mutations typically range from 0.85% to 6.7% (Kelliher et al., 2017; Liu et al., 2017; Gilles et al., 2017). This gene is highly conserved among cereal crops (Liu et al., 2020; Cheng et al., 2021; Tang et al., 2023). Subsequent studies demonstrated that knockout of its rice ortholog (*OsMATL*) successfully induced *in vivo* haploid formation with a haploid induction rate of 2%–6% (Yao et al., 2018), paving the way for the application of haploid breeding technology based on this mechanism in rice.

Recently, a number of advancements have been achieved in *in vivo* DH technology using haploid inducers. This review focuses on the latest progress in four key technical steps: haploid induction, haploid identification, chromosome doubling, and the cultivation/application of DH lines. The challenges and issues associated with each step are discussed, along with the potential applications of this technology in rice breeding.

## 2 Exploration of haploid induction genes in rice and development of high-efficiency rice haploid inducers

**Figure 1:**
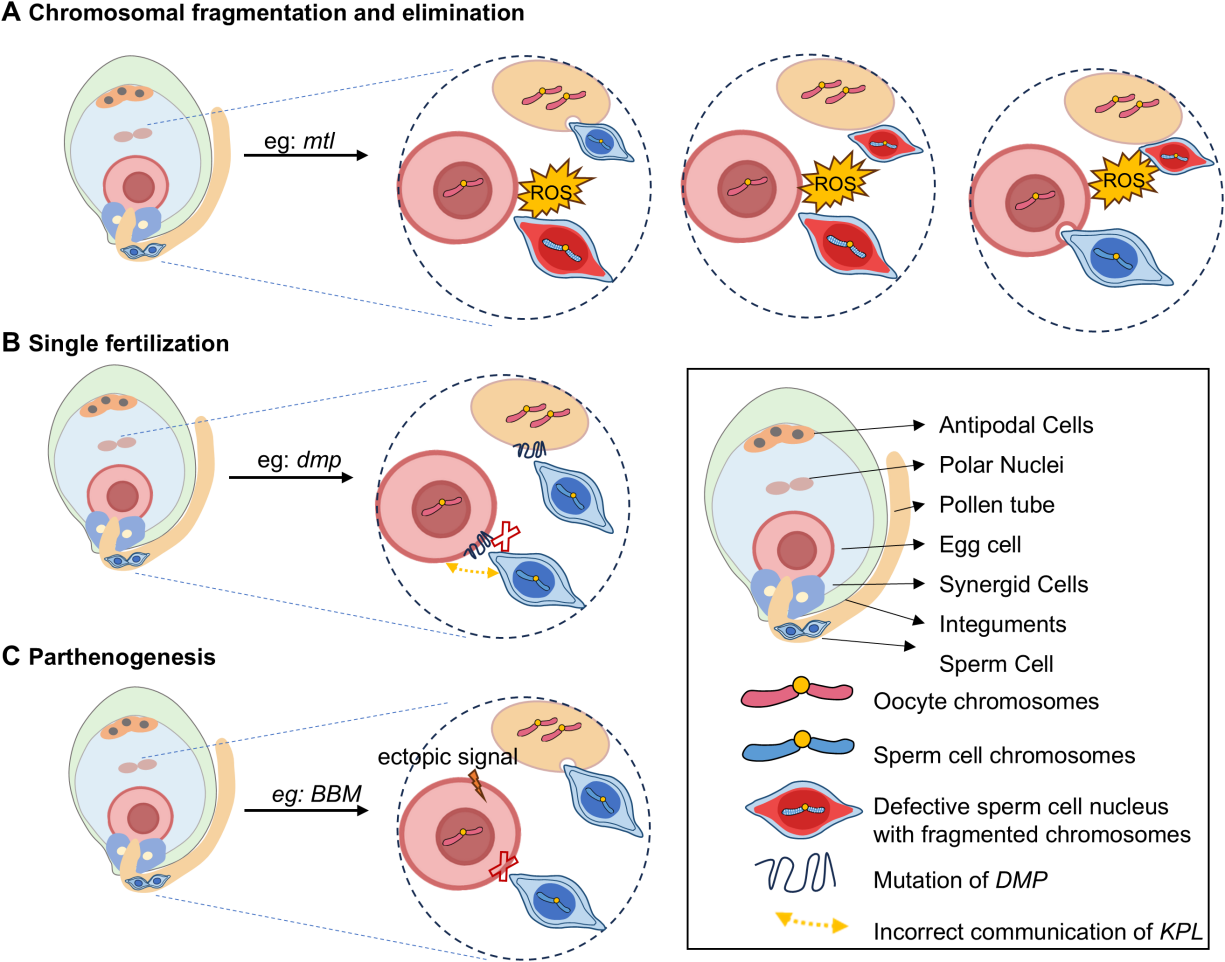
Schematic diagram of the underlying mechanisms of haploid induction in plants. Haploid induction occurs through three distinct mechanisms: uniparental genome elimination via sperm DNA fragmentation is primarily mediated by ROS overaccumulation. Following fertilization, paternal chromosome degradation occurs due to DNA damage, ultimately inducing maternal haploid formation **(A)**, In single fertilization, disrupted communication and fusion between male and female gamete plasma membrane, unfertilized egg cells develop into haploid individuals **(B)**, ectopic expression of embryogenic activators results in autonomous oocyte activation and production of paternal genome-free haploids **(C)**. This figure was created with Microsoft PowerPoint 2019.

Haploid induction represents the initial step of *in vivo* DH technology. Investigating the genes responsible for haploid induction is essential, as it provides a foundation for DH technology and facilitates the development of haploid inducers. In recent years, a number of genes involved in haploid induction have been identified in plants (**Table 2**). These genes are primarily associated with double fertilization, and disruption of their expression can impair normal fertilization, resulting in haploid offspring containing only one set of chromosomes from a parent. Based on the mechanisms of haploid induction, the related genes can be categorized into three main types: (i) uniparental genome elimination, (ii) single fertilization, and (iii) induction of parthenogenesis (**Figure 1**). Additionally, depending on how inducers are used, these genes can be grouped into two types: (i) haploid production through hybridization with inducers, and (ii) haploid production via self-pollination after crossing with inducers.

**Table 2:** Summary of reported haploid induction genes in plants.

Mechanism	Gene	Species	HIR (%)	Applicable in rice	Reference
Uniparental genome elimination	*IG1*	Maize	0.03	NR	Evans (2007)
	*CENH3*	Maize	5	No	Bortiri et al. (2024)
		*Arabidopsis*	25-66.7		Warburton et al. (1997); Yang et al. (2025)
		Wheat	7		Lv et al. (2020)
	*NLD/MTL/PLA1*	Maize	6-10	Yes	Gilles et al. (2017); Kelliher et al. (2017); Liu et al. (2017)
		Rice	2-12.4		Yao et al. (2018)
		Barley	12.8-16.4		Tang et al. (2023)
	*PLD3*	Maize	0.85–0.96	Yes	Li et al. (2021)
		Rice	0.3-0.6		Hu et al. (2025b)
	*POD65*	Maize	1.0-7.7	NR	Jiang et al. (2022)
	*pPLAIIγ*	*Arabidopsis*	1.07	Yes	Jang et al. (2023b)
		Rice	6.34		Jang et al. (2023a)
	*OspPLAIIκ*	Rice	0.23–0.31	Yes	Liu et al. (2025)
	*KNL2*	Maize	4.94	NR	Li et al. (2025c)
Single Fertilization	*ECS1/ECS2*	*Arabidopsis*	0.8-1.1	NR	Zhang et al. (2023)
	*DMP*	Maize	0.1-0.3	No	Zhong et al. (2019)
		Rice	0		Liu et al. (2024d); Hu et al. (2025a); Liang et al. (2025)
		Cucumber	0.09-0.40		Yin et al. (2024)
		Barrel medic	0.29- 0.82		Wang et al. (2022b)
		Oilseed rape	2.4		Zhong et al. (2022)
		Cotton	1.06		Long et al. (2024)
		Tobacco	1.2		Zhong et al. (2022)
	*KPL*	*Arabidopsis*	0.07-0.34	NR	Jacquier et al. (2023)
	*GEX1*	Maize	1.34	NR	Sun et al. (2025)
Parthenogenesis	*PsASGR-BBML*	Pearl millet	0-50	Yes	Conner et al. (2015)
		Maize	/		Conner et al. (2017)
		Rice	/		Conner et al. (2017)
		Tobacco	0.2-27.3		Zhang et al. (2020)
	*PAR*	Rice	0.59-1.02	Yes	Xiong et al (2025a; 2025b)
		Lettuce	13.3-25.6		Underwood et al. (2022)
		Foxtail millet	1.4-10.2		Huang et al. (2024)
	*BBMs*	*Arabidopsis*	0.28-6.74	Yes	Liu et al. (2024c)
		Rice	3.2-32.5		Khanday et al. (2019); Wei et al. (2023)
		Sweet potato	7.1		Song et al. (2025)
	*WUS*	Rice	/	Yes	Huang et al. (2025)
	*GEX1*	Maize	1.34	NR	Sun et al. (2025)

“/” indicates that the literature mentions haploid induction capability but provides no supporting data; “Yes” indicates applicable in rice (functional validation reported); “No” indicates no haploid induction efficiency observed in rice; “NR” (No Report) indicates not yet reported in rice.

### 2.1 Haploid induction via uniparental genome elimination

Uniparental genome elimination is a major mechanism for haploid induction. Haploids produced via this pathway do not inherit any genetic material from the haploid inducer. Several genes have been implicated in this process, including *MTL*, *pPLAIIγ*, *PLD3*, *CENH3*, *KNL2*, *POD65*, and *IG1*, among others.

The pollen-specific phospholipase gene *MTL* (also referred to as *ZmPLA1* or *NLD*) is the primary genetic determinant underlying haploid induction in maize (Kelliher et al., 2017; Liu et al., 2017; Gilles et al., 2017). A 4-bp insertion within *ZmMTL* has been identified as the causative mutation responsible for initiating haploid induction (Kelliher et al., 2017; Liu et al., 2017; Gilles et al., 2017). Research suggests that haploid induction mediated by *MTL* occurs due to chromosome fragmentation in the gametophyte after meiosis (Li et al., 2017), a phenomenon that may be linked to an abnormal burst in reactive oxygen species (ROS) during the later stages of pollen development (Jiang et al., 2022; Sun et al., 2022). *ZmMTL* is highly conserved across cereal crops, and knockout of its orthologs in both rice and wheat has been shown to induce haploid formation (Yao et al., 2018; Sun et al., 2022). Specially, genome editing of *OsMTL* in rice leads to higher HIR and seed setting rates (SSR) in *indica* varieties compared to *japonica* varieties. For example, in *indica* rice, HIR and SSR reached 6% and ~20% in IR58025B, 2.65% and 27.0%-35.1% in MingHui 63, and 2.65% and 20.3%-22.9% in HuaHang 48 (Yao et al., 2018; Liu et al., 2024d; Liang et al., 2025). In contrast, *japonica* varieties such as YanDao 8 (HIR: 1.4%; SSR: 13.2%) and Nipponbare (HIR: 1.3%; SSR: 6.7%-7.1%) exhibited lower values (Liang et al., 2025). However, these reported HIR levels are insufficient for practical application in DH technology breeding, which usually requires rates exceeding 7% (Wang et al., 2022a). Recently, a high-efficiency haploid inducer line named HI285 was developed in rice through the introgression of the *OsMTL* mutation into different genetic backgrounds, achieving an HIR of 11.8%-15.1% (Liu et al., 2024a).

The *MTL* gene belongs to the *phospholipase A* (*PLA*) family, which includes dozens of other members. Consequently, since the function of *MTL* was identified, researchers have investigated whether other proteins in this family also possess the ability to induce haploid formation. In *Arabidopsis*, knockout of *pPLAIIγ*—a pistil-specific member of the phospholipase gene family—can induce maternal haploids at an efficiency of 1.07% (Jang et al., 2023a). Importantly, knockout of its ortholog in rice, *OsMATL2* (also known as *OspPLAIIη* or *OspPLA-IIα*), which exhibits pollen-specific expression, produces haploid offspring at a rate of 6.34% (Jang et al., 2023b). Subsequent study have further revealed that OspPLAIIκ, a gene closely related to MTL within the PLA family, also possesses haploid induction capability, albeit at a lower frequency ranging from 0.23% to 0.31%. Notably, combining mutations in OspPLAIIκ with mtl did not lead to enhanced haploid induction rates, underscoring the complex functional relationships among these paralogs (Liu et al., 2025). Additionally, *Phospholipase A* (*PLA*), *phospholipase C* (*PLC*), and *phospholipase D* (*PLD*) all belong to the phospholipase gene family. Genes from other subfamilies have also been found to induce haploids. For example, mutation of *ZmPLD3*, a member of the *PLD* family and highly expressed in mature anthers and pollen grains, can induce haploids at a frequency of 0.85%–0.96% while maintaining a high seed-setting rate (Li et al., 2021). Furthermore, *zmpld3* exhibits synergistic effects with *mtl* in enhancing the HIR. Similarly, the rice ortholog *OsPLDα2* was identified and found to induce haploids at a frequency of 0.8%-1.2% without compromising fertility (Hu et al., 2025b). However, whether *OsPLDα2* and *OsMTL* exhibit synergistic effects in rice requires further investigation.

Other genes involved in uniparental genome elimination include *CENH3*, *KNL2*, *POD65*, and *IG1*. CENH3 (centromere-specific histone H3) is essential for centromere assembly and spindle attachment, ensuring accurate chromosome segregation during cell division. In *Arabidopsis*, specific modifications to CENH3 can induce haploids via chromosome elimination (Ravi and Chan, 2010; Marimuthu et al., 2011). KNL2, a CENH3-interacting protein, has also been shown to induce haploidy (Li et al., 2025c). Notably, high-temperature treatment during pollination enhances the haploid induction efficiency of both CENH3 and KNL2. Although *CENH3* is highly conserved in crops and haploid inducer lines based on *cenh3* have been developed in wheat and maize (Lv et al., 2020; Wang et al., 2021), no such success has been reported in rice. Additionally, mutations in *IG1* (*indeterminate gametophyte1*), which regulates the expression of proliferation-promoting genes in the embryo sac, can induce androgenic or paternal haploids at a rate of approximately 3% in maize (Evans, 2007). Mutation in *ZmPOD65*, encoding a pollen-specific peroxidase involved in ROS scavenging, disrupts ROS clearance in sperm cells, leading to ROS accumulation, DNA fragmentation, and haploid induction (Jiang et al., 2022). However, no orthologs of these genes have yet been reported to induce haploids in rice.

### 2.2 Haploid induction via single fertilization

Mutations in genes involved in the fertilization process—such as *DMP*, *ECSs*, *GEX1*, and *KPL2*—can be an approach to trigger haploid formation in plants. These genes primarily induce haploids through a single fertilization mechanism.

*ZmDMP* encodes a membrane protein containing a DUF679 domain and plays a key role in maize haploid inducers such as CAUHOI and CAU5 (Zhong et al., 2019). It is expressed in mature pollen and localized to the cell membrane. When combined with *ZmMTL* mutations, *ZmDMP* significantly enhances the HIR. Studies in *Arabidopsis* have shown that mutations in *DMP* induce haploid formation by interfering with fertilization and disrupting plasma membrane communication (Cyprys et al., 2019). Specifically, AtDMP8/9 interact with HAP2/GCS1—a key fusogen essential for sperm-egg membrane fusion and gamete recognition—and GEX2, a pollen-specific transmembrane protein involved in sperm cell adhesion. This interaction facilitates the translocation of HAP2/GCS1 to the sperm cell membrane in an EC1-dependent manner, ensuring proper gamete fusion (Wang et al., 2022c). Mutations in *DMP* disrupt this process, leading to failed gamete fusion, single fertilization, and consequently, haploid induction. Furthermore, functional orthologs of *ZmDMP* have successfully induced maternal haploids in several dicot species, including alfalfa (Wang et al., 2022b), cucumber (Yin et al., 2024), cotton (Long et al., 2024), oilseed rape, and tobacco (Zhong et al., 2022). However, *DMP* mutations have not been shown to induce haploids in monocots such as rice or wheat (Liang et al., 2025).

ECSs (egg cell-specific aspartic endopeptidases) play a critical role in ensuring the fusion of male and female nuclei after fertilization. They are also involved in preventing multiple pollen tubes from entering the embryo sac (Mao et al., 2023). In *Arabidopsis*, the *ecs1*/*ecs2* double mutant can use as a maternal haploid inducer, producing haploids at a frequency of 0.8%-1.1% (Zhang et al., 2023). Evidence suggests that haploid induction in this mutant is likely mediated by single fertilization. Moreover, low temperature significantly enhances haploid induction rates mediated by *ECS* (Zhao et al., 2025). In rice, genome editing of the orthologous *ecs1*/*ecs2* gene, *OsECS*, can also trigger haploid induction, achieving a frequency of 6.7% (Zhang et al., 2023).

In addition to *DMP* and *ECSs*, *GEX1* and *KPL* are two other genes known to induce haploids via single fertilization in plants. *GEX1* encodes a nuclear membrane protein specifically expressed in the egg and central cells of female gametophytes and plays a role in gametophyte development and early embryogenesis in *Arabidopsis* (Alandete-Saez et al., 2011; Nishikawa et al., 2020). In maize, *ZmGEX1* was identified through GWAS as being associated with fertility traits. Mutation of *ZmGEX1* does not affect normal embryo sac development but significantly impairs the fertilization process, ultimately leading to the induction of maternal haploids (Sun et al., 2025). The *KPL* gene, expressed in *Arabidopsis* male gametophytes, has also been shown to confer haploid-inducing ability when mutated (Jacquier et al., 2023). Its functional impairment may disrupt plasma membrane communication between male and female gametes, thereby inhibiting normal double fertilization. However, whether the orthologs of *GEX1* and *KPL* in rice exhibit similar haploid induction capabilities remains to be investigated.

### 2.3 Haploid induction via egg cell ectopic expression of parthenogenesis-related genes

Haploids can also be produced by the ectopic expression of genes implicated in parthenogenesis or associated with parthenogenesis-related pathways within the egg cell. Notable examples of such genes include *PsASGR*-*BBML*, *BBMs*, *PAR*, *WUS*, among others.

*PsASGR-BABY BOOM-like* (*PsASGR-BBML*) is the first identified parthenogenesis gene cloned from *Pennisetum squamulatum* (Conner et al., 2015). It shares structural similarities with the AP2 transcription factor family. Expressing *PsASGR-BBML* under its native promoter or egg cell-specific promoters can trigger parthenogenesis in several species, including sexual grasses (*Pennisetum glaucum*), tobacco (*Nicotiana tabacum*), rice, and maize (Conner et al., 2015; Zhang et al., 2020). In rice, using either the *Arabidopsis AtDD45* promoter or the native *PsASGR*-*BBML* promoter to drive the full-length gene results in more effective haploid embryo induction than using the native promoter to drive only the coding sequence of *PsASGR*-*BBML* (Dan et al., 2024).

Homologs of *PsASGR-BBML*, known as *BABY BOOM* (*BBM*) genes, are AP2/ERF transcription factors expressed in sperm cells and involved in embryogenesis (Boutilier et al., 2002). In rice, both ectopic expression of *OsBBM1* and *OsBBM4* in egg cells, driven by the *Arabidopsis AtDD45* promoter, can induce parthenogenesis. *OsBBM1* achieved a haploid induction rate of 5.8%-10.5% in the T_0_ generation, rising to 32.5% in T_1_, while *OsBBM4* showed a 3.2% induction rate with a seed-setting rate of 21.1%-82.6% (Khanday et al., 2019; Wei et al., 2023). Moreover, co-expression of *OsWOX9A* and *OsBBM1* in egg cells increased parthenogenesis to 86%-91%, up to 15-fold higher than *OsBBM1* alone (Ren et al., 2024). In maize, ectopic expression of *ZmBBM2* in egg cells has been reported to induced parthenogenesis with a maximum rate of 3.6% (Qi et al., 2023). In dicots, egg cell-specific expression of *BBM* driven by the *EC1* promoter successfully induced parthenogenesis and haploid embryo development in *Arabidopsis*, *Brassica napus*, sweet potato, and tomato (Chen et al., 2022; Liu et al., 2024c; Song et al., 2025).

The *PARTHENOGENESIS* (*PAR*) gene, identified in the naturally apomictic dandelion (*Taraxacum officinale*), encodes a unique C2H2 zinc finger domain and exhibits egg cell-specific expression. When expressed under the *Arabidopsis AtDD45* promoter, *ToPAR* induced parthenogenesis in both dandelion and lettuce (*Lactuca sativa*), producing haploid progeny at frequencies of 7.1% and 13.3-25.6%, respectively (Underwood et al., 2022). In rice, ectopic expression of *ToPAR* and *PpPAR* in egg cells also induced parthenogenesis, with HIR of 0.59-1.02% for *ToPAR* and 0.5-1.5% for *PpPAR* (Xiong et al., 2025a; 2025b). Furthermore, ectopic expression of *ToPAR* in foxtail millet (*Setaria italica*) driven by the *AtDD45* promoter achieved a maximum haploid induction rate of 10.02% (Huang et al., 2024). However, whether orthologs of *PAR* in other species possess similar haploid induction capabilities remains unknown.

Other genes, such as *WUSCHEL* (*WUS*), have also been associated with parthenogenesis. *WUS* overexpression enhances somatic embryogenesis and shoot regeneration in *Arabidopsis* and other species (Zuo et al., 2002; Bouchabké-Coussa et al., 2013). When combined with the *MiMe* (*mitosis instead of meiosis*), egg cell-specific expression of *OsWUS* enables the formation of clonal hybrid seeds in rice. In contrast, expression of *OsWUS* driven solely by the *AtDD45* promoter leads to dwarfism and sterilit (Huang et al., 2025).

### 2.4 Categorizing haploid induction genes by application strategy

**Figure 2:**
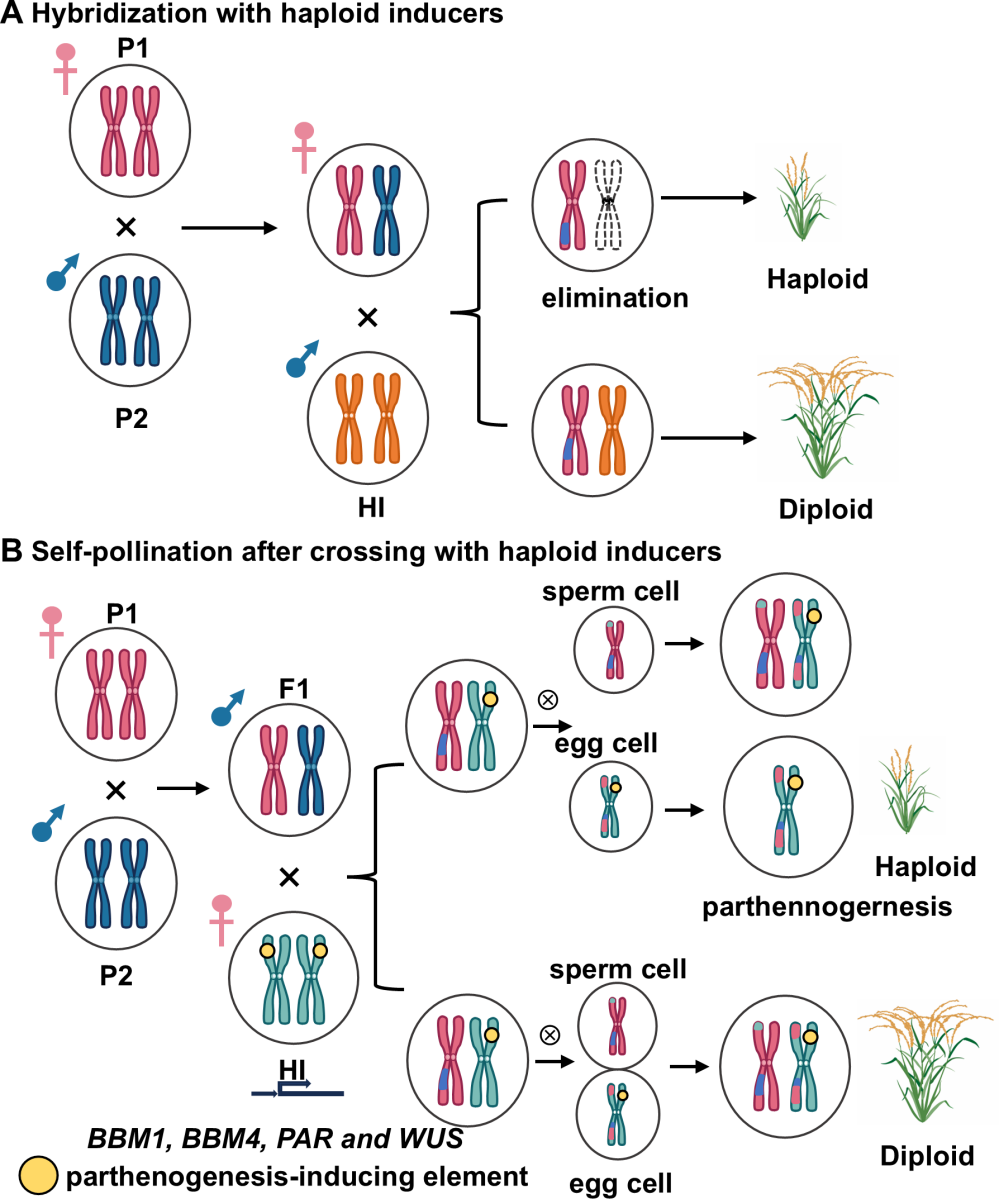
Approaches for producing haploids using different haploid induction genes. Haploid production can be achieved through hybridization with haploid inducers **(A)**, as well as through self-pollination after crossing with haploid inducers **(B)**. This figure was created with Adobe Illustrator 2021 (rice plants graphics) and Microsoft PowerPoint 2019.

Although a number of haploid induction genes have been identified in plants and can be classified into the three aforementioned types based on their underlying mechanisms, not all of them have been widely adopted in DH technology for breeding. Therefore, depending on their application strategies, we categorize haploid induction genes into two main types: (i) Haploid production through hybridization with inducers involved in uniparental genome elimination and single fertilization. A prominent example is the use of high-efficiency haploid inducers in maize, such as *MTL* and *DMP*. When utilizing these inducers, they are employed as the male parent and crossed with heterozygous lines. The resulting haploids contain genetic material exclusively from the female parent, with no genetic contribution from the haploid inducer. (ii) Haploid production via self-pollination following crossing with inducers mediated by parthenogenesis genes, such as *OsBBM1*, *OsBBM4*, and *PAR* (**Figure 2**). In this strategy, heterozygous lines are first crossed with haploid inducers. Subsequently, among the self-pollinated progeny, a subset of haploids can be identified. However, these haploids may carry ectopic expression elements—such as the *AtDD45* promoter—which could pose limitations for breeding applications. Based on these principles, we suggest that the *OsMTL*-mediated DH technology holds greater application potential in rice.

## 3 Rapid identification of haploids using morphological and molecular markers

After haploid induction, identifying haploids is essential before chromosome doubling. Common methods to distinguish haploids from diploid rice plants include morphological observation, flow cytometry, chromosome counting, high-throughput sequencing, morphological markers, and molecular markers (Ghalagi et al., 2023). Among these, flow cytometry, chromosome counting, and high-throughput sequencing are the most accurate, but they are labor-intensive, time-consuming, and costly, which limits their use in large-scale screening. Although haploids show morphological differences compared to diploids—such as shorter plant height, fewer tillers, shorter panicles, and sterile pollen—these traits usually appear only at later growth stages (Dermail et al., 2024). Early identification of haploids at the seed or seedling stage reduces the number of plants that need to be grown, saving labor, resources, and costs, and supporting efficient chromosome doubling. Therefore, developing morphological or molecular markers for early haploid identification would significantly improve the efficiency of DH technology.

**Figure 3:**
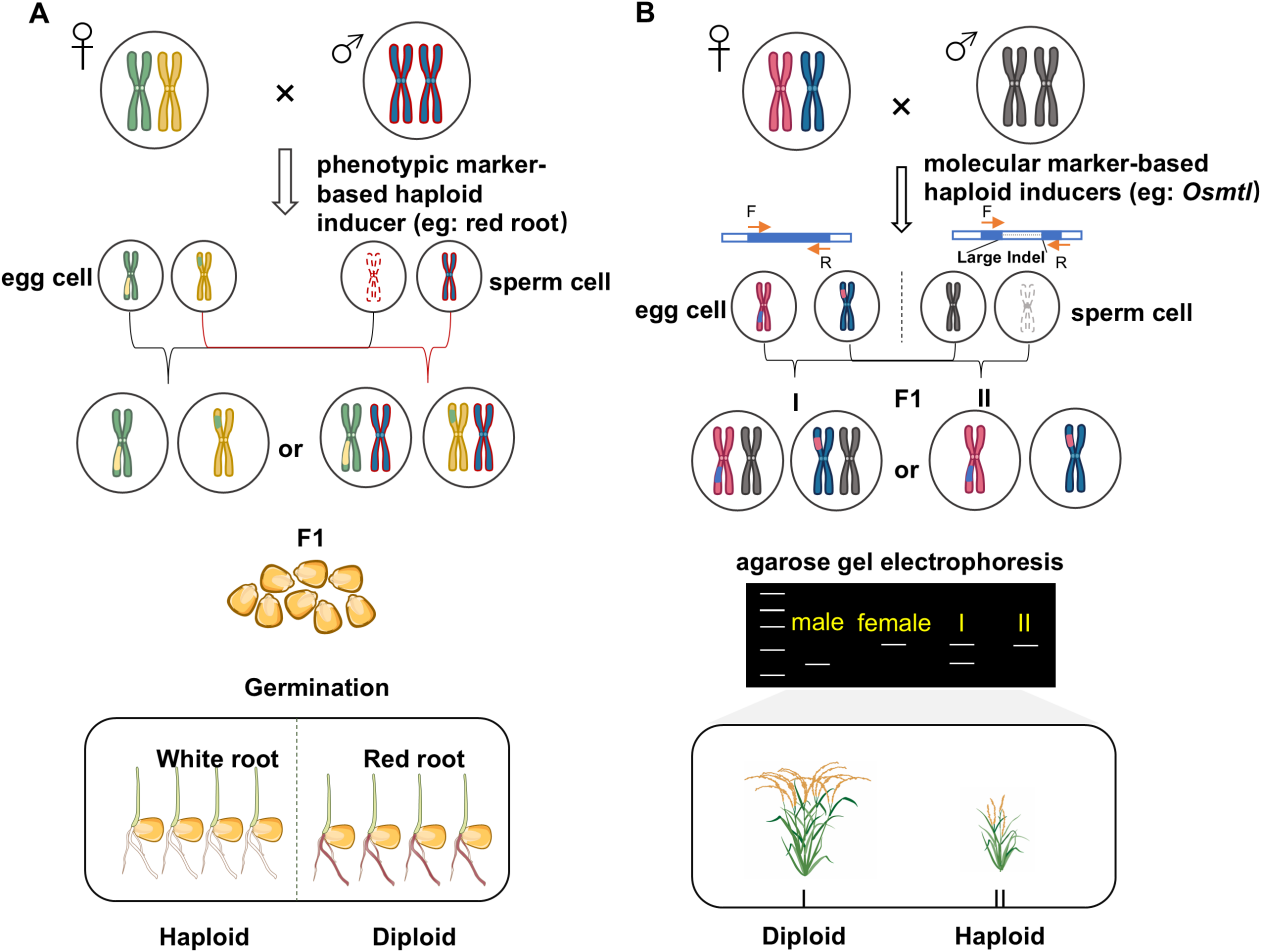
Haploid identification through marker-based haploid inducers. Workflow for haploid identification using specific morphological markers **(A)** and InDel markers **(B)**. This figure was created with Adobe Illustrator 2021 (rice plants graphics) and Microsoft PowerPoint 2019.

The principle of identifying haploids using morphological or molecular markers relies on their genetic difference: haploids contain only maternal chromosomes, while diploids have genomes from both parents (**Figure 3**). Thus, molecular markers can be designed to distinguish between them, enabling accurate identification at the rice seedling stage. When combined with rapid DNA extraction, this method becomes highly efficient. However, its effectiveness depends on specific breeding materials; each change in material requires redesigning markers. When multiple materials are used simultaneously, marker development becomes complex. Moreover, this method requires reference genomes, limiting its use for unsequenced wild rice or newly mutagenized lines. To address these issues, Wang et al. (2023b) used gene-editing to create large deletions or insertions in the *OsMTL* gene and designed primers around the edited site as an InDel marker. This marker is independent of the maternal genotype, works across rice varieties, and can be applied to maize and wheat. Notably, the high-efficiency haploid inducer line HI285 also contains such edits, allowing the development of universal InDel markers suitable for any breeding material.

Although InDel markers are useful in rice DH technology, they still require sampling, DNA extraction, PCR, and electrophoresis, making them less convenient for field breeders. In contrast, visual morphological markers offer a more user-friendly alternative. Maize benefits from well established visual markers including *R1-nj*, *MAGIC*, high-oil content, red root, fluorescent proteins (e.g., GFP, YFP), and the recently developed *RUBY* system (Melchinger et al., 2013; Chaikam et al., 2015, 2016; Chen et al., 2022; Wang et al., 2023a). While *R1-nj* is widely used, its efficacy can be affected by pigmentation inhibitors. Improved accuracy has been achieved through engineered lines, such as those overexpressing *ZmC1* and *ZmR2* for purple embryo identification (99.1% accuracy), or incorporating the *RUBY* marker for betalain-based detection across tissues and specie (Chen et al., 2022; Wang et al., 2023a). In rice, visual markers are less advanced, though recent systems using leaf tip morphology or glabrous leaves show promise for early and low-cost screening, despite being limited by genetic specificity (Ghalagi et al., 2023). Future integration of visible markers like *RUBY* into efficient inducer lines (e.g., HI285) could significantly streamline haploid identification. Additionally, AI-assisted technologies—such as NMR/NIR-based oil content detection and computer vision for automated color scoring—hold great potential for high-throughput haploid screening (Song et al., 2017).

## 4 Chromosome doubling of haploid plants through colchicine treatment

After induction and identification, haploids must be chromosomally doubled to become doubled haploids. Current doubling methods include spontaneous and artificial approaches, with low and variable spontaneous doubling rates limiting efficient DH production. Although some haploids can produce fertile pollen spontaneously, the frequency is generally low and highly genotype- and environment-dependent. In maize, spontaneous doubling rates range from 1% to 70% (Chaikam et al., 2019a). In rice, spontaneous doubling rates range from 50% to 60% (Seguí-Simarro and Nuez, 2008). Genome-wide association studies (GWAS) have identified associated loci, but no functional genes have been cloned, and the mechanism remains unclear. Further genetic studies are needed to elucidate the molecular basis and potential applications of spontaneous doubling.

Artificial doubling relies mainly on chemical or physical methods. Colchicine, which inhibits spindle formation and arrests mitosis, is the most widely used doubling agent and accounts for 40% of rice doubling protocols (Singh et al., 2023). However, it is highly toxic, leading to plant loss during treatment, poor viability of doubled plants, and risks to human health and the environment. Recently, less toxic alternatives with comparable efficiency—such as flufenacet, oryzalin, and trifluralin—have been developed (Chaikam et al., 2019b). Key factors affecting doubling efficiency include reagent type, concentration, exposure time, temperature, and plant developmental stage. For instance, combining DMSO and Tween 20 improves reagent penetration and doubling rates (Eliby et al., 2022; Kaur et al., 2023). Excessive concentration or prolonged treatment reduces survival, and an inverse relationship exists between antimitotic concentration and duration (Sabzehzari et al., 2019). A temperature of 25 °C is optimal for colchicine-treated plant recovery. In barley, treatment at the four-tiller stage yielded 90% DHs, compared to 56% at the 2–3 leaf stage (Subrahmanyam and Kasha, 1975; Jensen, 1976).

**Figure 4:**
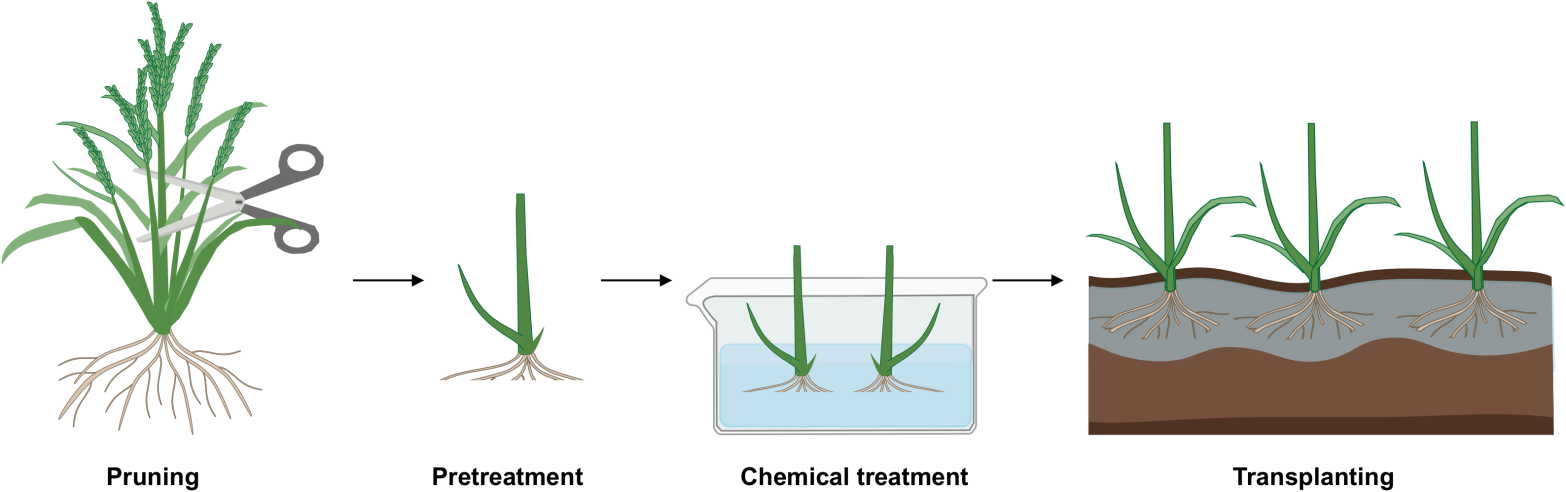
Schematic diagram of chromosome doubling of haploid plants in rice. The key steps of haploid chromosome doubling primarily include: cutting the plant base 5–10 cm above ground during heading; after 2–3 days, uprooting and cleaning the plants, removing leaf sheaths while keeping lateral buds (2–3 cm long); soaking in a solution of 2% DMSO and 0.1% colchicine for 24 hours; then rinsing and transplanting to the field. This figure was created with Adobe Illustrator 2021 (vector graphics) and Microsoft PowerPoint 2019.

A standard protocol for chromosome doubling is as follows (Liu et al., 2024a). The collected haploid plants are first rinsed with tap water. Subsequently, the roots and shoots were trimmed to approximately 2 cm and 10 cm in length, respectively. The plants were then placed in a shaded area to promote the formation of new buds. After new buds emerged, plants with aligned root bases were bundled and transferred into 2-L round plastic containers. A solution of 50 μmol/L oryzalin (or 0.1% colchicine) containing 2% DMSO was added to each container until the roots and new buds were fully submerged. The treatment was carried out at room temperature for 12 to 24 hours. After treatment, the spent chemical solution was collected in labeled toxic waste containers for safe disposal. Finally, the plants were thoroughly rinsed with tap water three times before being transplanted into well-irrigated field plots under standard agronomic management (**Figure 4**).

Physical pretreatments such as cold or heat shock applied before *in vitro* culture can disrupt cytoskeletal components and induce doubling. Irradiation (γ-rays, X-rays, UV-C) can also double chromosomes by damaging spindle microtubules or DNA repair pathways but may cause mutations. Nitrous oxide (N_2_O) is a gaseous colchicine alternative that inhibits tubulin polymerization. It yielded doubling rates comparable to colchicine in maize under 0.6 MPa pressure, without toxicity or waste disposal issues (Molenaar et al., 2018). However, initial equipment costs limit its widespread adoption. Notably, no studies have specifically reported the application of physical treatments for chromosome doubling in rice to date. Given the advantages of physical treatments (e.g., avoiding chemical residues associated with colchicine) and their proven efficacy in other plants, exploring their feasibility, optimal parameters, and molecular mechanisms for chromosome doubling in rice represents a promising avenue for future research.

## 5 Applications of DH technology in rice improvement

### 5.1 Synthetic apomixis

The integration of haploid induction with the *MiMe* (*mitosis instead of meiosis*) system enables synthetic apomixis for fixing heterosis in rice. For example, simultaneous knockout of *MiMe* genes (including *OsPAIR1*, *OsREC8*, and *OsOSD1*) along with *OsMTL* or *OsPLDα2* using CRISPR-Cas9 technology allows the production of clonal seeds in hybrid rice (Wang et al., 2019; Liu et al., 2023; Liu et al., 2024b; Hu et al., 2025b). Furthermore, genome editing of *MiMe* genes combined with ectopic expression of parthenogenesis-related genes—such as *OsBBM1*, *OsBBM4*, *PpPAR*, *ToPAR*, and *OsWUS*—in egg cells has successfully engineered synthetic apomictic systems exhibiting high seed set or clonal propagation rates (Khanday et al., 2019; Wei et al., 2023; Song et al., 2024; Huang et al., 2025; Xiong et al., 2025b).

### 5.2 HI-Edit and IMGE

The HI-Edit (Haploid Inducer-Edit) platform combines haploid induction with CRISPR/Cas9-mediated editing to enable rapid and transgene-free trait introgression (Kelliher et al., 2019). Originally developed in maize, this system has been used to introduce targeted edits in recalcitrant inbred lines such as B73 at the *ZmLG1* locus. By co-delivering a haploid induction cassette, fluorescent markers, and CRISPR components, HI-Edit has successfully generated knockout mutations in genes such as *Wx1* and *Sh2*, achieving high maternal haploid induction rates (8.55–20.89%) across multiple commercial lines (Li et al., 2025b). Similarly, the IMGE (Haploid Inducer-Mediated Genome Editing) strategy incorporates CRISPR/Cas9 into a haploid inducer line, which is then crossed with elite materials to produce edited doubled haploid progeny. Although HI-Edit has been validated in species such as maize, Arabidopsis, wheat, and cabbage, its application has not yet been reported in rice (Li et al., 2025a, 2025).

### 5.3 Generation of cytoplasmic male-sterile lines

DH technology significantly accelerates the development of CMS lines through rapid cytoplasmic substitution. In maize, a cenh3-based haploid inducer carrying a dominant seed marker is crossed with a CMS cytoplasm donor to create a CMS-conversion stock (Bortiri et al., 2024). This stock is then used as a female in crosses with a restorer-deficient elite line. Haploid seeds carrying the recurrent nuclear genome and the CMS cytoplasm are selected and subjected to chromosome doubling, resulting in homozygous CMS lines within two generations—bypassing the need for lengthy backcrossing. A parallel approach has been applied in broccoli, where introgression of a *cenh3* mutant into a CMS donor enabled direct transfer of the Ogura cytoplasm into elite inbreds, streamlining hybrid seed production (Han et al., 2024). In rice, DH technology also facilitates the development of Photo-Thermosensitive Genic Male Sterile (PTGMS) lines (Liu et al., 2024a).

## 6 Current challenges and future perspectives

Rice DH technology currently faces four major bottlenecks: (a) no haploid inducer line combines both a high HIR and elite agronomic traits; (b) self-pollination restricts large-scale hybridization; (c) a rapid, high-throughput, and cost-effective haploid identification method is lacking; and (d) a cost-effective, less toxic and efficient chromosome doubling protocol is still needed. Despite these limitations, DH technology holds significant potential to accelerate breeding cycles.

To facilitate the application of DH technology in rice breeding programs, several strategies should be considered. First, emphasis should be placed on mid- to low-generation heterogeneous materials, where phenotypic pre-screening allows the selection of desirable segregants prior to haploid induction. Advanced homozygous lines offer limited gains from DH technology, and the use of F_1_ hybrids can introduce excessive genetic variability. Second, priority should be given to implementing the technology in two-line male sterile lines, as conventional inbreds and restorer lines are less amenable, and three-line CMS systems remain incompatible. The inherent facultative male sterility in rice can be leveraged to generate heterozygous sterile populations without the need for emasculation. For conventional varieties, thorough emasculation is critical to minimizing false positives. Finally, integrating DH technology with other advanced tools—such as gene editing and RiceNavi—represents a promising strategy to improve trait precision and enhance genetic gain.
